# Mindful mamas: Black and Latina mothers’ mindful parenting predicts toddlers’ later social–emotional and cognitive functioning

**DOI:** 10.1017/S0954579425101004

**Published:** 2025-12-17

**Authors:** Lindsay Taraban, Julia S. Feldman, Pamela A. Morris-Perez, Alan L. Mendelsohn, Daniel S. Shaw

**Affiliations:** 1 Department of Psychiatry, University of Pittsburgh, Pittsburgh, PA, USA; 2 Department of Applied Psychology, Education and Human Development, New York University Steinhardt School of Culture, New York City, NY, USA; 3 Department of Pediatrics, New York University School of Medicine, New York, NY, USA; 4 Department of Psychology, University of Pittsburgh, Pittsburgh, PA, USA

**Keywords:** early childhood, language development, maternal depression, mindful parenting, social-emotional development

## Abstract

This study examined longitudinal associations between maternal mindful parenting and child social–emotional, behavioral, and language development. Maternal mindful parenting at 18 months was tested for associations with concurrent observed maternal responsivity and lack of punishment toward the child and as a predictor of child internalizing symptoms, externalizing symptoms, social competence and productive language 6 months later, independent of maternal depressive symptoms (a known predictor of both parenting and child outcomes). We also tested whether child negative emotionality (NE) moderated associations between mindful parenting and child outcomes. Participants (*N* = 316 mothers) were low-income (mean annual income = $19,024), racially and ethnically diverse mothers (48.4% Black; 43.0% Latinx) recruited from Pittsburgh, PA and New York City, NY. Higher mindful parenting was concurrently associated with higher observed maternal responsiveness toward the child and longitudinally associated with all four child outcomes in expected directions; maternal depression was a significant predictor of child internalizing and externalizing symptoms. Contrary to hypotheses, at moderately high levels of child NE, the positive effects of mindful parenting on child outcomes were attenuated. Results provide preliminary evidence that mindful parenting is meaningfully associated with parenting behaviors and early childhood developmental outcomes above and beyond symptoms of maternal depression.

## Introduction

### Mindfulness and parenting

In the context of Western psychological theory, mindfulness has been defined as “the awareness that emerges through paying attention on purpose, in the present moment, and non-judgmentally to the unfolding of experience” (Kabat-Zinn, 2003; p. 145). In contrast to the disciplined mindfulness mediation practices associated with Eastern spiritual traditions (Schmidt, 2011), mindful attention is an accessible skill that reduces automatic and often unhelpful responses to everyday life experiences (Brown et al., 2007; Duncan et al., 2009). Applied to parenting, mindfulness may support a strong parent – child relationship via parents’ ability to “pay attention to their child in a way that is intentional, present-centered, and non-judgmental” (Coatsworth et al., 2018; Duncan et al., 2009). As detailed in Huynh et al. (2024), mindful parenting shares conceptual overlap with parental reflective functioning (i.e., mentalizing), in that both concepts involve parental engagement in cognitive and affective processes to understand the thoughts and feelings of self and child in the service of healthy emotional co-regulation. Despite their overlap, mindful parenting and reflective functioning are thought to be distinct constructs. For example, reflective functioning requires the parent to consider the impact of past experiences in shaping their emotional reactions, while mindful parenting focuses on parents’ allowing difficult emotions to arise while staying grounded in the present moment (Huynh et al., 2024).

Duncan and colleagues (Duncan et al., 2009) proposed one of the most commonly used frameworks for mindful parenting, which includes listening to the child with full attention, appreciating the child, nonjudgmental acceptance of the child, self-regulation and emotional awareness in the parenting context, and compassion for self and child. From a theoretical perspective, it is easy to appreciate how a parent who listens closely, can stay calm when their child is dysregulated, and demonstrates compassion in parent – child interactions (including challenging ones), would foster positive child social – emotional and behavioral development. As an example, in the context of a young child’s temper tantrum, a parent with low levels of mindful attention may experience a great deal of negative emotion (e.g., frustration, anxiety) and respond reflexively with anger or withdrawal, increasing both the child’s distress in the moment and the likelihood of their future dysregulation. On the other hand, high mindful attention would aid a parent in remaining calm and confident during a child’s tantrum, allowing them to accurately assess their child’s needs and provide appropriate support, with expected positive downstream effects on the child’s longer-term emotional and behavioral regulation.

Much of the work examining mindful parenting has focused on adolescents and utilized concurrent measurement of mindful parenting and youth outcomes. The vast majority of research has used self-report of mindful parenting (for an exception, see Geier, 2012). Within predominately White samples in the U.S., higher self-reported mindful parenting has been concurrently associated with lower parent-reported child internalizing and externalizing problems among children aged 3 – 17 years (Parent et al., 2016), and with lower adolescent-reported substance use (Turpyn & Chaplin, 2016). Utilizing a longitudinal design, Coatsworth et al. (2018) found that fathers’ (but not mothers’) self-reported mindful parenting was associated with decreases in youths’ (69% White; 6^th^ and 7^th^ graders) self-reported aggression across a one-year period. Among international samples, higher self-reported mindful parenting has been concurrently associated with lower adolescent-reported internalizing symptoms (Dutch sample; Geurtzen et al., 2015), lower adolescent-reported emotional problems (Mainland Chinese sample; Wang et al., 2018), and higher youth-reported overall well-being (Portuguese sample; child ages 8 – 19; Medeiros et al., 2016).

A smaller number of studies have explored associations between self-reported mindful parenting and child outcomes during infancy. Mindful parenting has been concurrently associated with lower levels of infant internalizing problems in an Australian sample (Burgdorf & Szabó, 2020) and positive infant temperament among a U.S. sample (Gartstein, 2021). Exploring such associations longitudinally, Dutch mothers (*N* = 90) who reported higher mindfulness during pregnancy also reported fewer problems with self-regulation and negative reactivity in their 10-month-old infants (van den Heuvel et al., 2015). Among a U.S. sample (*N* = 73; 77% White), higher mindful parenting at 3-months postpartum predicted a steeper cortisol recovery slope for infants at 6-months-postpartum during a stressful task, suggesting more adaptive stress reactivity (Laurent et al., 2017).

Although existing evidence suggests an association between mindful parenting and child emotional and behavioral outcomes, there is a dearth of longitudinal research examining associations between mindful parenting and child developmental outcomes during infancy and the toddler period, developmental periods where associations between parenting and child outcomes are known to be particularly strong (Landry et al., 2008; Lovejoy et al., 2000). In addition, with one exception (see Rivera et al., 2022), all samples exploring associations between mindful parenting and child outcomes within North America have been composed primarily of White participants. The present study begins to address these gaps by testing whether maternal mindful parenting at child age 18 months longitudinally predicted child social, emotional, behavioral, and cognitive development at 24 months, among a sample of primarily Black and Latina mothers living in urban communities in the United States.

### Self-reported mindful parenting and observed parenting behaviors

Mindful parenting has been theorized to impact child outcomes through its connections to a variety of positive caregiving practices, such as warmth, appreciation, and responsivity (Duncan et al., 2009). Within the U.S., mothers (*N* = 375) in the top quartile of self-reported mindfulness demonstrated more positive observed parenting (e.g., lower harshness, greater warmth) during a discussion task with their adolescent children compared to mothers in the bottom quartile (Duncan et al., 2015). Among a Chinese sample of mother – adolescent dyads (*N* = 168), maternal self-reported mindful parenting was concurrently associated with greater observed expressed warmth (Wang et al., 2018). Additionally, in one of the few studies with very young children, Dutch mothers of infants and toddlers participated in a 9-week mindfulness intervention. Mothers displayed higher levels of sensitivity and acceptance toward the child following participation in the intervention (compared to pre-intervention observations), suggesting that the mindfulness intervention had a positive impact on parenting practices (Potharst et al., 2017; Zeegers et al., 2019).

Despite some empirical support for a link between self-reported mindful parenting and observed parenting behaviors, there is no prior work in North American samples exploring such associations among racially and ethnically diverse parents or among parents of young children. There is, however, some evidence that parenting behaviors tend to differ across cultures, although it is important to acknowledge that a great deal of variability exists within a given racial or ethnic group, and broad generalizations may not be particularly useful. That said, research has indicated that Black parents may use a stricter, more hierarchical, or more authoritarian style with their young children compared to White parents (for a review, see Jambunathan et al., 2000). Findings for Latinx parents have been mixed, with some studies reporting higher levels of permissiveness and others reporting higher authoritarianism compared to their White counterparts (Jambunathan et al., 2000). Regardless, we should not assume that findings related to mindful parenting and observed parenting would be equivalent across racial and ethnic groups. Thus, the current study sought to examine whether Black and Latina mothers’ reports of mindful parenting were concurrently associated with observed maternal responsivity and lack of punishment toward their young children.

### Mindful parenting and maternal depression

To further probe associations between self-reported mindful parenting and child outcomes, we included control for one of the most frequently used and well-researched maternal measures of well-being: maternal depressive symptoms. There are several established, brief screeners for parental depressive symptoms [e.g., Center for Epidemiologic Studies Depression Scale (CES-D), Edinburgh Postnatal Depression Scale (EPDS), Patient Health Questionnaire-9 (PHQ-9)] which are frequently used in clinical, research, and medical settings (Earls et al., 2019; Lovejoy et al., 2000). Importantly, maternal depressive symptoms have been routinely linked to child outcomes both concurrently and longitudinally, including child internalizing and externalizing problems, and cognitive development (for a review, see Goodman et al., 2011) and are also associated with observed maternal parenting (e.g., sensitivity, warmth; for a review, see Taraban & Shaw, 2018). Further, maternal depressive symptoms have been negatively associated with maternal mindful parenting among mothers of infants (Fernandes et al., 2021a; Nobre-Trindade et al., 2021).

Thus, we were interested in controlling for maternal depression based on its established association with all our variables of interest (i.e., mindfulness, observed parenting, child outcomes). Determining whether self-reported mindful parenting predicts later child outcomes above and beyond maternal reports of symptoms of maternal depression has important implications both for our conceptual understanding of self-reported mindful parenting and for the potential clinical utility of this measure should mindfulness continue to predict child outcomes after accounting for another measure also based on maternal report (i.e., minimizing variance due to method and/or informant bias).

### Mindful parenting and child outcomes: moderation by child negative emotionality

An exploration of associations between mindful parenting and child outcomes is incomplete without consideration of the child’s contribution to the relationship. Theoretically, it may be easier and more enjoyable to parent mindfully for a child who tends to be easygoing and happy compared to a child who is fussy and difficult to soothe. Negative emotionality (NE), a core component of temperament, refers to a child’s tendency to react to environmental stressors with high levels of emotionality, including irritability, anger, sadness, and fear (Paulussen-Hoogeboom et al., 2008; Rothbart et al., 2000). In one prior study, Portuguese mothers who perceived their infant as having a difficult temperament reported higher parenting stress and lower mindful parenting compared to mothers who perceived their infant as being easygoing (Fernandes et al., 2021b). In addition, previous research has found high child NE in early childhood to be associated with presumed correlates of lower mindful parenting, including lower levels of parental warmth and sensitivity as well as child emotional and behavioral problems (Crockenberg & Leerkes, 2003; Engle & McElwain, 2011; Paulussen-Hoogeboom et al., 2008).

Although high child NE may be associated with lower levels of mindful parenting overall, for children high on NE who nonetheless have a mother who is able to parent with high mindful attention, we may expect mindful parenting to have a particularly positive impact on development. Children who are high on NE are likely both more dependent on their caregivers for help with emotion regulation and present a greater number of opportunities for their caregiver to respond to emotional distress, as well as being at higher risk for emotional and behavioral problems. Thus, just as parental monitoring (protective factor) has been found to be more strongly associated with adolescent development for adolescents living in higher- versus lower-risk communities (Herman et al., 2020), mindful parenting may have a particularly positive impact on development for children high on NE compared to children who become upset less often and are better able to self-soothe (i.e., low NE). This expectation is consistent with the differential susceptibility hypothesis, which posits that certain individuals are both more susceptible to adversity *and* to the beneficial effects of an enriched environment (Belsky & Pluess, 2009; Roisman et al., 2012). In particular, child NE has been identified as an important factor for differential susceptibility, with multiple studies finding that the association between various parenting practices (e.g., positive and negative parenting, discipline) and child outcomes are stronger for children high on NE (for a review, see Belsky, 2005). Thus, we expected high child NE to increase the strength of associations between mindful parenting and child problem and prosocial behaviors, while we expected low child NE to attenuate these associations.

### Current project

Prior literature provides promising preliminary results suggesting that higher mindful parenting may contribute to positive parenting practices (e.g., higher levels of observed warmth, sensitivity; e.g., Duncan et al., 2015; Wang et al., 2018; Zeegers et al., 2019) and positive child emotional and behavioral development (e.g., Geurtzen et al., 2015; Medeiros et al., 2016; Parent et al., 2016). The current project extends the current literature by testing longitudinal associations between mindful parenting and child social, emotional, behavioral, and cognitive development during early childhood among a racially and ethnically diverse group of U.S.-based mothers and their children, who were part of a randomized controlled trial aimed at improving early school readiness. Intervention effects have been reported in prior publications, with the primary findings being that the intervention was effective at improving children’s cognitive outcomes and reducing behavior problems at ages 4 (language, literacy) and 6 (language, literacy, math, cognition), with most effects being mediated through improvements in parental cognitive stimulation and reductions in harsh parenting (Canfield et al., 2025; Miller et al., 2024, 2023; Roby et al., 2021). As the intervention was not a focus of the present study, we controlled for intervention status in all analyses. We also considered maternal depressive symptoms and child NE in understanding associations between mindful parenting and child outcomes. The specific aims and hypotheses of the current project were as follows:

1. Test direct associations of maternal self-reported mindful parenting at 18 months with observations of maternal parenting (responsivity, lack of punishment) at 18 months, and child outcomes at 24 months (internalizing symptoms, externalizing symptoms, social competence, productive language), controlling for concurrent maternal depressive symptoms. We expected mindful parenting to be associated with greater observed maternal responsivity and lack of punishment toward the child. We also expected higher levels of mindful parenting to predict lower levels of child internalizing and externalizing symptoms, and higher levels of child social competence and productive language.
2. Test child NE at 18 months as a moderator of the associations between maternal mindful parenting at 18 months and all four child outcomes at 24 months. We expected that the association between mindful parenting and child outcomes would be stronger for children with higher levels of NE.

## Method

### Participants

Participants came from the Smart Beginnings Study, a sample of mothers and infants (*N* = 403) recruited from birthing hospitals (and then primary care clinics) between 2015 and 2017 in New York City and Pittsburgh, PA shortly following the child’s birth (Roby et al., 2021). Eligibility criteria included being on public insurance; full-term singleton birth with no significant medical problems, developmental delays, or disabilities; mother as primary caregiver; and mother being fluent in either English or Spanish. Participants were included in the current project if mothers completed the measure of mindful parenting at the 18-month assessment (*n* = 316).

Similar to the larger Smart Beginnings sample, the analytic sample was low-income and racially and ethnically diverse, with roughly a third primiparous (first time birth) mothers (*n* = 110). Most mothers identified as either African American (*n* = 142, 45%) or Latina (*n* = 138, 44%). The remaining mothers identified as Asian (*n* = 4, 1%), White/Caucasian (*n* = 22; 7%), or other (*n* = 10, 3%). Seventy percent of mothers had received a high school diploma or equivalent (*n* = 217), with 34% of those also completing at least some college (*n* = 105). In terms of partnership status, 79% of mothers were married or cohabitating with the child’s biological father at the time of the child’s birth (*n* = 249). The average annual income of mothers at the time of the child’s birth was $19,024 (*SD* = 15,530). Mothers were an average of 28 years old at the birth of the target child (*SD* = 5.7 years). Thirty-four percent of mothers (all at the New York City site) spoke Spanish as their primary language. Forty-six percent of the children were female. Similar to the racial and ethnic background of mothers, the children were primarily African American (47%) or Latinx (43%). *Chi*-square and independent samples *t*-tests revealed no significant differences on sociodemographic or study variables between the analytic subsample and families excluded from analyses with one exception: children included in the present study were less likely to be female than children excluded from analyses, *χ* ^2^ (1) = 9.64, *p* < .01. In terms of attrition, at age 24 months, 287 mothers (91% of analytic sample) responded to the child outcome questionnaires. *Chi*-square and independent samples *t*-tests revealed that there were no significant differences on sociodemographic or study variables between the retained and attrited groups.

### Procedure

This study involved secondary analysis of data from the Smart Beginnings project. The Smart Beginnings project is a randomized controlled intervention trial focusing on parenting and children’s school readiness outcomes (Miller et al., 2023; Roby et al., 2021). Families assigned to the intervention condition (*N* = 201; 49.8%) were offered PlayReadVIP (i.e., formerly known as Video Interaction Project) intervention (PR-VIP; Mendelsohn et al., 2018), aimed at improving early cognitive development through parent – child play. PR-VIP participation involved meeting with a bachelor’s level PR-VIP coach during their child’s regularly scheduled pediatric primary care visits beginning shortly after birth. During the 30-minute PR-VIP sessions, parents received a developmentally appropriate toy or book and were videotaped playing or reading with their infant for several minutes, with the coach identifying strengths in the interaction and providing feedback about additional opportunities for interaction. Intervention families who were particularly high risk, based on sociodemographic, family, and child factors, were also offered a second intervention, the Family Check-Up (FCU; Dishion et al., 2014) at 6 months (*N* = 90; 22%) and again at 18 months (*N* = 126; 31%). The FCU is an individually tailored, strengths-based intervention, aimed at improving parenting skills to address child behavior problems. The FCU consists of an initial interview, an assessment, and a feedback session. Following the feedback session, parents could engage in evidence-based parent management training with the family coach (Dishion et al., 2014). Control group families (*N* = 202; 50%) were not offered any intervention, but like intervention families continued to receive well-child visits at their pediatric home. As the intervention was not a focus of the present study, intervention status was included as a covariate in all analyses. See Roby et al. (2021) for additional details regarding the Smart Beginnings project intervention. IRB approval for the Smart Beginnings Study (“Integrated model for promoting parenting and early school readiness in pediatrics”) was obtained from The University of Pittsburgh, and New York University School of Medicine.

Data for the present study came from assessments at child ages 18 and 24 months. During these assessments, which were scheduled to last approximately 2 hours, mothers completed questionnaires on a wide range of topics pertaining to themselves and their babies and participated in a series of interaction tasks with their baby. Mothers were informed that the purpose of the visit was to learn about how the child was developing and how things were going in the home and in the mother’s life. Mothers were asked to minimize distractions during the visit (e.g., turn off television, silence phone) and were encouraged to tend to their child’s needs prior to beginning research activities (e.g., diaper change, feeding, nap). A sitter was available to tend to other children who might be present. Parent – child tasks were completed first, to minimize burden on the child, followed by parental completion of questionnaires. The 24-month assessment visit took place in the lab. The 18-month assessment was meant to take place in the family’s home, and in Pittsburgh, most visits (86%) were home visits. The feasibility of conducting home visits in New York proved more challenging because of difficulties scheduling families in their homes, and concerns about travel time and neighborhood safety. Thus, in New York, slightly more than half of the total 18-month visits were conducted in the lab (57%). The same protocol was used for home and lab visits.

### Measures

#### Mindful parenting

Maternal mindful parenting was measured during the 18-month assessment via maternal report on the Interpersonal Mindfulness in Parenting Scale (IM-P; Duncan, 2007). The IM-P has been validated among several culturally diverse samples (e.g., Chinese mothers, Portuguese mothers; (Duncan et al., 2015; Lo et al., 2018; Moreira & Canavarro, 2017), and among mothers of infants (Burgdorf & Szabó, 2020). To keep the assessment as brief as possible for the purposes of the larger study, two subscales from the 31-item measure thought to be most relevant for child outcomes during infancy and the toddler period were administered, as decided by the study team. These included the *listening with full attention* factor (5 items; e.g., “I find myself listening to my child with one ear because I am busy doing or thinking about something else”), and the *emotional awareness of self and child* factor (5 items; e.g., “how I am feeling tends to affect my parenting decisions, but I do not realize it until later”; Duncan 2023). Mothers used a five-point scale from *Never True (0)* to *Always True (4)* to respond to each statement as it applied to their daily interactions with their child. Traditionally, higher scores on the IM-P represent lower levels of mindfulness. For ease of interpretation in the present study, items were reverse coded so that higher scores represented higher levels of maternal mindfulness. These items were then summed and averaged to create the mindfulness score for each mother. A Spanish version was administered for Spanish-speaking mothers. Following best practices, the English version of the IM-P was translated, back-translated, and then reviewed by Spanish-speaking members of the team to ensure that the meaning of the items was retained. Internal consistency for the 10-item measure in the present sample was acceptable (total, *α* = .78; English, *α* = .78; Spanish, *α* = .78).

#### Maternal depressive symptoms

Maternal depressive symptoms were measured during the 18-month assessment using the Edinburgh Postnatal Depression Scale (EPDS; Cox et al., 1987). The EPDS has been found to be sensitive to changes in depression over time and has been validated to screen for depression in mothers beyond the postpartum period (Cox et al., 1996). The EPDS includes ten statements, to which participants respond on a scale ranging from 0 (*no, not at all*) to 3 (*yes, quite a lot*), with higher scores representing greater symptoms of depression within the past 7 days. Sample items include, “I have blamed myself unnecessarily when things went wrong,” “I have been anxious or worried for no good reason,” and “I have been so unhappy that I have had difficulty sleeping.” A previously validated Spanish translation of the EPDS was used for Spanish-speaking families. Internal consistency on the EPDS at 18 months in our sample was acceptable (total, *α* = .81; English, *α* = .83; Spanish, *α* = .78).

#### Observed Maternal parenting

Impressions of mothers’ interactions with their children at 18 months were captured using observationally based items from the Infant – Toddler Home Observation Measure of the Environment (IT-HOME; Bradley, 2012). The IT-HOME has been found to be a valid and reliable measure among samples drawn from high-risk populations, including low socioeconomic status and maternal psychopathology, and variations by race and ethnicity (Bradley, 2014; Bradley & Corwyn, 2002; Bradley et al., 1994). At the conclusion of each 18-month home assessment, research assistants who had conducted the visit completed the 11 binary items that comprise the IT-HOME Responsivity Subscale (e.g., *parent responds verbally to child’s vocalizations; parent spontaneously praises child at least twice;* total, *α* = .86; home visits, *α* = .70; lab visits, *α* = .80), and 5 out of 6 binary items that comprise the IT-HOME Acceptance/Lack of Punishment Subscale (e.g., *parent does not express overt annoyance with child; parent does not interfere or restrict child more than 3 times;*). The full Acceptance/Lack of Punishment Subscale from the IT-HOME includes one additional question that is answered through parental interview (i.e., instances of physical punishment in the past week), but consistent with other research, items included in the present study included only those that could be observed during the research visit (for a review, see Fuligni et al., 2004). Research assistants responded to IT-HOME items using a *Yes* (1)/*No* (0) response format, with higher scores on each subscale representing higher levels of positive parenting behaviors. As these subscales were examiner impressions and coded by only one observer, inter-rater reliability is not available.

#### Child negative emotionality

Child NE was measured at 18 months via maternal report on the Infant – Toddler Social and Emotional Assessment (ITSEA; Carter et al., 2003). The ITSEA has been found to have acceptable test – retest reliability and validity among diverse groups of parents of young children (Carter et al., 2003). The current study used the 13-item NE subscale, which asks mothers to report on how true each statement is about their child using the anchors *Not True/Rarely (0), Somewhat True/Sometimes (1),* and *Very True/Often (2)*. Sample items include, “Cries if doesn’t get own way,” and “Able to wait for things” (reverse scored). A previously validated Spanish version of the ITSEA was used for Spanish-speaking families. Internal consistency for this subscale in the present sample at 18 months was good (total, *α* = 0.85; English, *α* = .85; Spanish, *α* = .85).

#### Child internalizing and externalizing symptoms

During the 24-month assessment, mothers completed the Preschool Version of the Child Behavior Checklist (CBCL), designed for children ages 1.5 to 5 years-old (Achenbach & Rescorla, 2000). The Preschool Version of the CBCL has demonstrated good test – retest and inter-rater reliability and has been validated among diverse groups of parents and children (Achenbach, 2015; Achenbach & Verhulst, 2010). The current study used the 35 items that comprise the externalizing problems subscale, and the 24 items that comprise the internalizing problems subscale. Parents used a 3-point scale ranging from *not true (as far as you know) (0),* to *very true/often true (2)*, to report on their child’s behavior within the past two months. Sample items from the externalizing subscale included “destroys things belonging to his or her family or to other children,” “gets in many fights,” and “physically attacks people.” Sample items from the internalizing subscale included “feelings are easily hurt,” “gets too upset when separated from parents,” and “too shy or timid.” A previously validated Spanish version of the CBCL was used for Spanish-speaking families. Internal consistency for each of the English and Spanish versions for the externalizing (English *α* = 0.91; Spanish *α* = .88) and internalizing (English *α* = 0.82; Spanish *α* = 0.88) subscales was good.

#### Child social competence

Child social competence was measured at 24 months using maternal report of the child on the Infant – Toddler Social and Emotional Assessment (ITSEA; Carter et al., 2003). Specifically, this study used 32 items from the *social – emotional competencies* domain, which is composed of 6 subscales: compliance, attention, imitation/play, mastery motivation, and empathy, and prosocial peer relations. Competencies are viewed as reflecting the presence of age-appropriate skills, rather than simply a lack of problem behaviors (Carter et al., 2003). Mothers rated how true each statement was of the target child, with the anchors *Not true/Rarely (0), Somewhat True/Sometimes (1),* and *Very True/Often (3)*. Examples of items include, “Enjoys challenging activities” and “Plays well with other children.” Internal consistency for the competence factor in this sample was good (total, *α* = .87; English, *α* = .87; Spanish, *α* = .88).

#### Child language

Children’s productive vocabulary was measured at 24 months using maternal report on the MacArthur Communicative Development Inventory – Short Form (CDI-SF; Fenson et al., 2000). The CDI-SF is widely used and has been found to have high validity and test – retest reliability (Fenson et al., 2000). The current study used the toddler version of the CDI-SF, which is appropriate for children 16 – 30 months of age. The CDI-SF asks mothers to report on their child’s productive vocabulary by checking off words the child can say. The list includes 100 words in various semantic categories, including nouns, sounds, action words, pronouns, words about time, and question words, among others. The English version of the CDI was translated into Spanish for the Spanish-speaking families, rather than using the Spanish version of the measure. This decision was made by the larger study team, as the Spanish version of the CDI uses different words than the English version and had been normed in Mexico, with children that did not share the cultural or demographic background of many in the current sample. For bilingual children, mothers reported on their child’s productive vocabulary of both English and Spanish words, and two scores were generated for each child. The *total score* is based on a simple addition of the English and Spanish words known, while the *conceptual score* reflects the total number of concepts the child produces words for across both languages. For example, a bilingual child who knows the words “shoe” and “zapato” would receive 2 points based on the total score and 1 point based on the conceptual score. Because we were interested in children’s full productive vocabulary, and because prior research has indicated that the language development of bilingual children is underestimated when they are tested in only one language (Hoff & Core, 2015) we used children’s total score on the CDI as our language outcome.

#### Covariates

Demographic covariates were collected from mothers as part of a structured interview that took place shortly after the child’s birth. The following covariates were included in all analyses: child race, child ethnicity, child gender, parental education, and intervention status. Intervention status was a binary variable which assessed whether the family was assigned to the intervention (*1*) or control condition (*0*). We originally also included study site (New York/Pittsburgh) as a covariate, but removed it due to high correlation between study site and child race/ethnicity (i.e., 81% of Pittsburgh sample was Black and 84% of New York sample was Latinx). Controlling for both site and child race/ethnicity did not meaningfully affect any results presented below.

### Data analytic plan

Descriptive statistics and bivariate correlations were conducted in SPSS (28). To test direct effects between maternal mindful parenting at 18 months, concurrent observed responsivity and lack of punishment, and child outcomes at 24 months within a multivariate framework (Aim 1), path analysis was conducted in Mplus (8.8; (Muthén & Muthén, 2017). Specifically, maternal mindful parenting (18 months) was modeled as a predictor of 18-month observed maternal parenting (responsivity and lack of punishment) and of 24-month child outcomes (internalizing symptoms, externalizing symptoms, social competence, language). To test whether associations between 18-month maternal mindful parenting and 24-month outcomes were moderated by 18-month child NE (Aim 2), maternal mindful parenting, child NE, and their interaction term were modeled as predictors of all four 24-month child outcomes. Using G*Power (version 3.1; (Faul et al., 2007), we determined, based on our sample size (*n* = 316), and using a two-sided test of alpha = .05 and requiring a power of .80, that we would be able to detect small effect sizes of *f* ^2^ = .024. In both the Aim 1 and Aim 2 models, maternal depressive symptoms, child race, child ethnicity, child gender, parental education, and intervention status were included as covariates and entered as predictors of primary variables. All longitudinal paths were estimated. Covariances among covariates were estimated only for variables with significant bivariate correlations. Continuous predictor variables and covariates were centered prior to analyses in the moderation models. Model fit was assessed using the *Chi*-square test (*p* > .05), comparative fit index (CFI > .90), and root mean square error of approximation (RMSEA < .05 for good fit and RMSEA < .08 for acceptable fit; (Mcdonald & Ho, 2002).

For Aim 2 moderation analyses, significant interactions were examined with simple slopes, using calculation tools available at quantpsy.org (Preacher et al., 2016). The strength of associations between maternal mindful parenting (1 standard deviation above and below the mean) and child outcomes were assessed at different levels of child NE (1 standard deviation above and below the mean, as well as the mean). *T*-tests were used to assess the significance of the simple slopes for each outcome of interest.

## Results

### Descriptive statistics and bivariate correlations

Descriptive statistics and bivariate correlations among primary study variables and covariates are presented in Tables 1 and 2, respectively. Maternal self-reported mindful parenting at 18 months was significantly correlated with all four 24-month child outcomes in expected directions. In comparison, maternal self-reported depressive symptoms at 18 months were significantly correlated with child internalizing and externalizing symptoms in expected directions but were not associated with child social competence or child language. Mindful parenting and depressive symptoms were significantly negatively associated with one another. In terms of concurrent parenting, mindful parenting was positively and significantly associated with observed maternal responsivity; maternal depressive symptoms were significantly negatively associated with observed maternal lack of punishment. Mindful parenting was significantly negatively associated with child NE, and child NE was significantly associated with all four child outcomes in expected directions.

**Table 1.** Descriptive statistics of primary study variables and covariates

Measure	Child Age	*n*	Range	Mean(SD) or *%*
Child Latinx	6 mo.	136		43%
Child Black	6 mo.	153		48%
Child White	6 mo.	8		3%
Child other race	6 mo.	14		4%
Child female	6 mo.	144		46%
Treatment family	6 mo.	153		48%
Maternal education	6 mo.	312	1–15	8.06(2.56)
Maternal mindful parenting	18 mo.	316	13–40	32.71(5.13)
Maternal depressive Sx	18 mo.	310	0–20	3.89(4.08)
Maternal responsivity	18 mo.	307	0–11	9.81(1.79)
Maternal lack of punishment	18 mo.	298	0–7	4.92(1.72)
Child negative emotionality	18 mo.	315	0–25	7.95(4.88)
Child internalizing symptoms	24 mo.	287	0–46	9.37(6.98)
Child externalizing symptoms	24 mo.	288	0–36	12.18(7.90)
Child social competence	24 mo.	288	17–64	44.61(8.99)
Child language	24 mo.	286	1–119	34.77(24.64)

**Table 2.** Bivariate correlations between primary study variables and covariates

Variable	1	2	3	4	5	6	7	8	9	10	11	12	13	14
Covariates (6 mo.)														
1. Child Latinx	–													
2. Child black	−.87**	–												
3. Child other race	−.19**	−.21**	–											
4. Child female	.06	−.08	.02	–										
5. Treatment family	−.03	.02	−.02	−.09	–									
6. Maternal education	−.37**	.26**	.18**	−.01	.11	–								
Mother measures (18 mo.)														
7. Mindful parenting	−.20**	.24**	−.05	.10	.03	.04	–							
8. Depressive Sx	−.15**	.16**	−.09	.05	−.06	.08	−.32**	–						
9. Responsivity	.08	−.10	.03	.04	.17**	.15*	.15*	.01	–					
10. Lack of punishment	.51**	−.53**	.09	.08	−.05	−.07	−.07	−.18**	.17**	–				
Child measures (24 mo.)														
11. Neg. emotionality	.01	.05	−.10	−.13*	.00	−.11	−.34**	.28**	−.21**	−.18**	–			
12. Internalizing	.31**	−.27**	−.09	−.04	−.06	−.29**	−.32**	.28**	−.17**	.09	.44**	–		
13. Externalizing	−.05	.05	−.06	−.10	−.01	−.02	−.30**	.42**	−.12*	−.18**	.48**	.55**	–	
14. Social competence	−.01	−.02	.09	.14*	.09	.17**	.21**	−.11	.24**	.07	−.23**	−.22**	−.32**	–
15. Language	−.10	.05	.10	.12	.00	.04	.17**	.02	.14*	.01	−.16**	−.19**	−.16**	.39**

*Note.* *p < .05, **p < .01.

The location of the study assessment (i.e., home visit versus lab visit) was not associated with significant differences on any self-reported or demographic predictor variables. Location of assessment was also not associated with differences in observed responsivity. However, families who had their 18-month visit in the lab had higher levels of observed lack of punishment (i.e., lower punishing behaviors; mean = 6.25, SD = 1.33) compared to families who had their 18-month visit in the home (mean = 4.34, SD = 1.49; *t* (301) = 10.77, *p* < .001).

### Aim 1: maternal mindful parenting: associations with concurrent observed responsivity and lack of punishment and prediction of subsequent child outcomes

**Figure 1.**
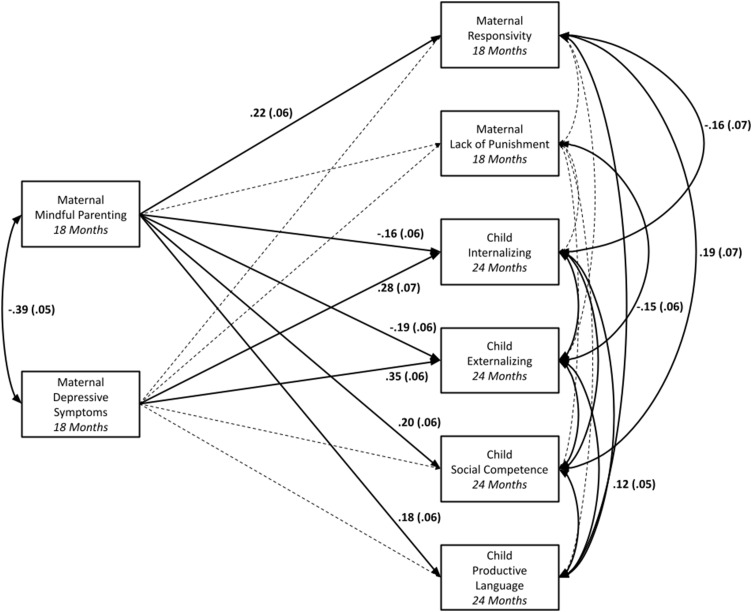
Aim 1 findings: Path analysis testing associations between mindful parenting (18 months), observed maternal responsivity and lack of punishment (18 months), and child internalizing symptoms, externalizing symptoms, social competence, and productive language (24 months), controlling for maternal depressive symptoms (18 months) and demographic covariates. *Note.* Solid lines represent significant associations; dashed lines represent nonsignificant associations. Demographic covariates not shown for ease of visualization.

Direct paths and covariances between primary variables (i.e., maternal mindful parenting and depressive symptoms, observed responsivity, lack of punishment, and child outcomes) can be found in Table 3. Direct paths and covariances with and between demographic covariates can be found in Supplemental Table 1. Model fit was excellent, χ^2^ (9) = 9.79, *p* > .36; RMSEA = .02, 90% CI [.00, .07]; CFI > .99. As expected, maternal mindful parenting was positively significantly associated with observed maternal responsivity. Unexpectedly, maternal mindful parenting was not associated with observed lack of punishment. Maternal depressive symptoms were not significantly associated with observed maternal responsivity or lack of punishment. As expected, maternal mindful parenting was significantly negatively associated with child internalizing and externalizing symptoms and significantly positively associated with child social competence and productive language. Maternal depressive symptoms were positively significantly associated with child internalizing and externalizing symptoms, but not with child social competence or productive language. Primary findings are displayed in Figure 1.

**Table 3.** Aim 1: path analysis testing associations between mindful parenting (18 months), observed maternal responsivity and lack of punishment (18 months), and child internalizing symptoms, externalizing symptoms, social competence, and productive language (24 months), controlling for maternal depressive symptoms (18 months), and demographic covariates

Path	β (SE)	*p*
**Mindful parenting **→** maternal responsivity**	**.22 (.06)**	< .001***
Mindful parenting → maternal acceptance	.02 (.05)	.76
**Mindful parenting **→** child internalizing**	**−.16 (.06)**	.01*
**Mindful parenting **→** child externalizing**	**−.19 (.06)**	.002**
**Mindful parenting **→** child social competence**	**.20 (.06)**	.001**
**Mindful parenting **→** child language**	**.18 (.06)**	.004**
Maternal depressive sx. → maternal responsivity	.10 (.07)	.15
Maternal depressive sx. → maternal lack of punishment	−.10 (.06)	.08^+^
**Maternal depressive sx. **→** child internalizing**	**.28 (.07)**	< .001***
**Maternal depressive sx. **→** child externalizing**	**.35 (.06)**	< .001***
Maternal depressive sx. → child social competence	−.03 (.06)	.60
Maternal depressive sx. → child language	.08 (.06)	.23
**Mindful parenting ←→ maternal depressive sx.**	**−.39 (.05)**	< .001***
Maternal responsivity ←→ maternal lack of punishment	.07 (.06)	.29
**Maternal responsivity ←→ child internalizing**	**−.16 (.07)**	.03*
Maternal responsivity ←→ child externalizing	−.09 (.07)	.19
**Maternal responsivity ←→ child social competence**	**.19 (.07)**	.004**
**Maternal responsivity ←→ child language**	**.12 (.05)**	.02*
Maternal lack of punishment ←→ child internalizing	−.03 (.06)	.66
**Maternal lack of punishment ←→ child externalizing**	**−.15 (.06)**	.02*
Maternal lack of punishment ←→ child social competence	.03 (.06)	.64
Maternal lack of punishment ←→ child language	.06 (.06)	.36
**Child internalizing ←→ child externalizing**	**.52 (.04)**	< .001***
**Child internalizing ←→ child social competence**	**−.13 (.05)**	.01*
**Child internalizing ←→ child language**	**−.16 (.05)**	.003**
**Child externalizing ←→ child social competence**	**−.26 (.06)**	< .001***
**Child externalizing ←→ child language**	**−.14 (.06)**	.01*
**Child social competence ←→ child language**	**.37 (.05)**	< .001***

*Note. N* = 316. ^+^ *p* < .10, *p < .05, **p < .01; ***p < .001.

### Aim 2: moderation of the association between maternal mindful parenting and child outcomes by child negative emotionality

**Figure 2.**
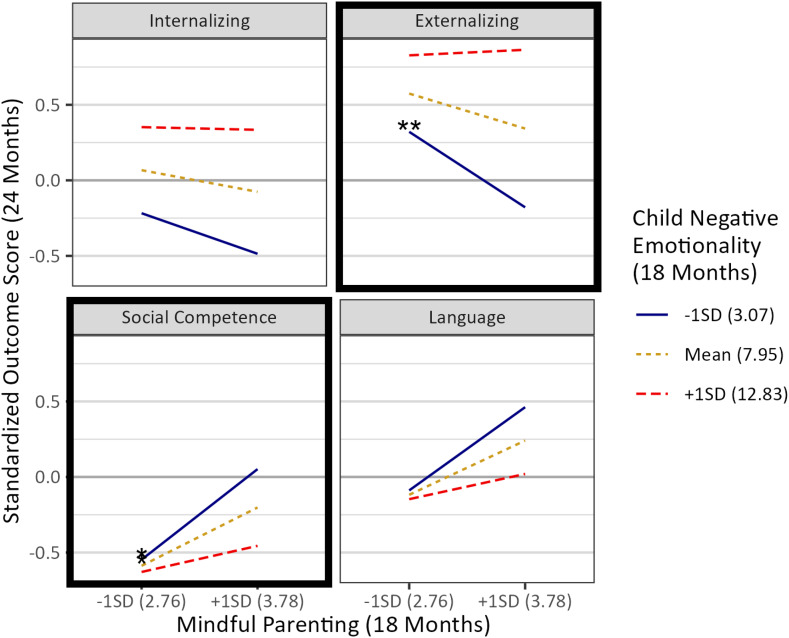
Association between mindful parenting (18 months) and child outcomes (24 months), moderated by child negative emotionality (18 months). *Note.* Child outcomes at 24 months are *z*-scored to facilitate interpretation. Significant interactions are indicated by bolded boxes. We note that the pattern of interaction was similar for all four outcomes, with mindful parenting being most strongly associated with the given child outcome at low levels of child negative emotionality and most weakly associated with the given child outcome at high levels of child negative emotionality. *indicates simple slope with *p* < .05. ** indicates simple slope with *p* < .01.

Child NE at 18 months was tested as a moderator of the associations between maternal mindful parenting and all four child outcomes at 24 months. Direct paths, moderated paths, and covariances between primary variables (i.e., maternal mindful parenting and depressive symptoms, child NE, and child outcomes) can be found in Table 4. Direct paths and covariances with and between demographic covariates can be found in Supplemental Table 2. Model fit was adequate, χ^2^ (18) = 36.86, *p* < .01; RMSEA = .06, 90% CI [.03, .08]; CFI = .96. Child NE significantly moderated the association between 18-month maternal mindful parenting and 24-month child externalizing symptoms and social competence. Child NE did not significantly moderate associations between maternal mindful parenting and child internalizing symptoms or productive language, although moderation for child internalizing approached statistical significance (i.e., *p* = .09). Simple slopes analyses revealed that children’s levels of externalizing symptoms at 24 months were negatively related to maternal mindful parenting for children with low levels of NE (i.e., defined as being one standard deviation below average), *B* = −3.84, *SE* = 1.30, *t* = −2.96, *p* < .01 (see Figure 2). Similarly, child social competence at 24 months was positively associated with maternal mindful parenting for children with low (one standard deviation below average), *B* = 5.14, *SE* = 1.81, *t* = 2.84, *p* = .01, or average levels of NE, *B* = 3.31, *SE* = 1.22, *t* = 2.71, *p* = .01 (Figure 2). Thus, contrary to hypotheses, high levels of child NE attenuated – rather than enhanced – associations between mindful parenting and child externalizing symptoms and social competence.

**Table 4.** Aim 2: path analysis testing child negative emotionality as moderator of relations between mindful parenting (18 months), and child internalizing symptoms, externalizing symptoms, social competence, and productive language (24 months), controlling for maternal depressive symptoms (18 months), and demographic covariates

Path	β (SE)	*p*
Mindful parenting → child internalizing	−.07 (.06)	.24
Mindful parenting → child externalizing	−.11 (.06)	.07
**Mindful parenting → child social competence**	**.19 (.07)**	.005**
**Mindful parenting → child language**	**.18 (.06)**	.005**
**Child negative emotionality → child internalizing**	**.35 (.06)**	< .001***
**Child negative emotionality → child externalizing**	**.38 (.05)**	< .001***
**Child negative emotionality → child social competence**	**−.15 (.07)**	.02*
Child negative emotionality → child language	−.12 (.06)	.05^+^
Mindful parenting × NE → child internalizing	.07 (.06)	.20
**Mindful parenting × NE → child externalizing**	**.15 (.05)**	.002**
**Mindful parenting × NE → child social competence**	**−.12 (.06)**	.03*
Mindful parenting × NE → child language	−.11 (.06)	.09^+^
**Maternal depressive sx. → child internalizing**	**.21 (.06)**	.001**
**Maternal depressive sx. → child externalizing**	**.27 (.06)**	< .001***
Maternal depressive sx. → child social competence	.00 (.06)	.99
Maternal depressive sx. → child language	.10 (.06)	.11
**Mindful parenting ←→ child negative emotionality**	**−.36 (.06)**	< .001***
**Mindful parenting ←→ maternal depressive sx.**	**−.39 (.05)**	< .001***
**Child negative emotionality ←→ maternal depressive sx.**	**.30 (.06)**	< .001***
**Child internalizing ←→ child externalizing**	.45 (.04)	< .001***
Child internalizing ←→ child social competence	−.09 (.05)	.097^+^
**Child internalizing ←→ child language**	**−.12 (.05)**	.02*
**Child externalizing ←→ child social competence**	**−.22 (.06)**	< .001***
Child externalizing ←→ child language	−.10 (.06)	.09
**Child social competence ←→ child language**	**.35 (.05)**	< .001***

*Note. n* = 315 (child NE not available for one family). ^+^ *p* < .10, *p < .05, **p < .01; ***p < .001.

### Exploratory analyses

**Figure 3.**
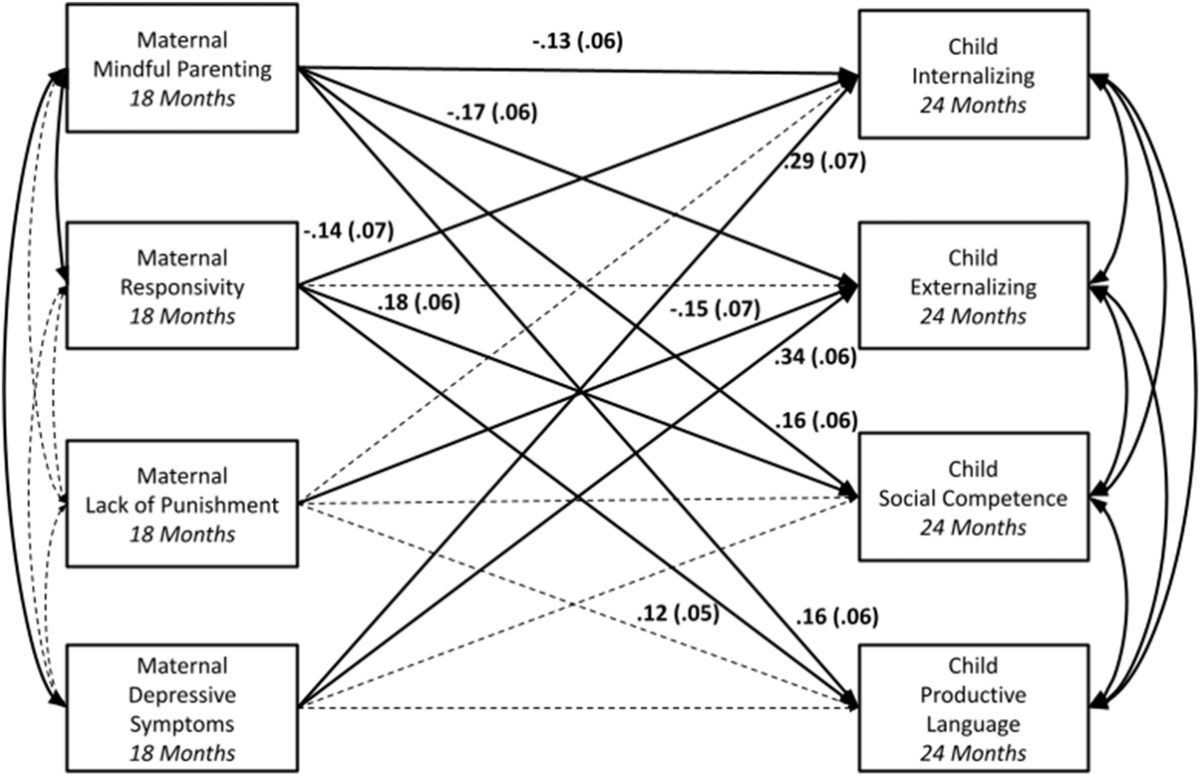
Post hoc exploratory analysis: path analysis adding the two observed parenting measures at 18 months (maternal responsivity, lack of punishment) as predictors of child internalizing symptoms, externalizing symptoms, social competence, and productive language (24 months), controlling for maternal depressive symptoms (18 months) and demographic covariates. *Note.* Solid lines represent significant associations; dashed lines represent nonsignificant associations. Demographic covariates not shown for ease of visualization.

We conducted two post hoc exploratory analyses to further probe the relationships among our variables of interest. First, we re-ran our Aim 1 model with the two observed parenting measures (maternal responsivity, lack of punishment) included as predictors of the child outcomes, along with maternal mindful parenting and symptoms of depression. In this model, maternal mindfulness continued to significantly predict all four child outcomes in expected directions, and maternal depression continued to predict child internalizing and externalizing symptoms in expected directions, consistent with findings for Aim 1. Maternal responsivity significantly predicted child internalizing, social competence, and language, and maternal lack of punishment predicted child externalizing symptoms (Figure 3; Supplemental Table 3). Second, we tested maternal depressive symptoms as a moderator of associations between mindful parenting and all four child outcomes. Maternal depression did not moderate any of these associations (Supplemental Table 4).

## Discussion

Among a low-income sample of Black and Latina mothers, we hypothesized that maternal mindful parenting at 18 months would be significantly associated with 18-month observed responsivity and lack of punishment and predict 24-month child social – emotional and language development (Aim 1). We found that maternal mindfulness – but not maternal depression – was a significant predictor of observed maternal responsiveness toward the child and further, that higher levels of maternal mindful parenting significantly predicted lower child externalizing symptoms and internalizing symptoms, and higher social competence and productive vocabulary, controlling for 18-month maternal depressive symptoms. Consistent with our Aim 2 hypothesis that child NE would moderate associations between mindful parenting and child outcomes, we did find support for this expectation for two out of four child outcomes; however, the pattern of moderation differed from our hypothesis such that high levels of child NE attenuated associations between mindful parenting and child outcomes.

### Maternal mindful parenting: links to early childhood outcomes

Mindful parenting at 18 months was a significant predictor of child internalizing symptoms, externalizing symptoms, social competence and language at 24 months, controlling for relevant demographic factors and symptoms of maternal depression. The mindfulness items utilized in the present study focused on mothers’ ability to remain present and engaged when interacting with their child and prevent their emotional reactions to the child from interfering with their parenting goals. Although the present study did not test mechanisms linking mindful parenting to child outcomes, classic theory (i.e., Ainsworth et al., 1978) suggests that when mothers pay close attention to their child and can stay calm during challenging interactions, this can help the child come to view the mother as a secure base and develop a secure attachment with them, which may contribute to the child’s feelings of safety and security, and thus, reduce the likelihood of internalizing symptoms. Empirical work has demonstrated this secure mother – child attachment also has a positive downstream effect on child social competence (Bohlin et al., 2000). Findings from the present study are also consistent with a prior study which found higher maternal mindful parenting as measured by the IM-P to be concurrently associated with lower levels of infant internalizing problems (Burgdorf & Szabó, 2020).

In terms of child behavioral problems, much research on early-emerging child disruptive behavior has focused on the “coercive cycle” (Patterson et al., 2004; Scaramella & Leve, 2004) that can develop between parents and children, in which child aversive behavior and parent negativity both increase in intensity over time. Theoretically, a highly mindful parent may be better equipped to avoid unwittingly reinforcing child oppositional behavior by modeling aggression. Instead, by remaining calm and compassionate in the face of child misbehavior, parents can respond appropriately by setting (and sticking to) reasonable limits and helping the child redirect to a more desirable behavior, reducing the likelihood of externalizing symptoms.

In terms of child cognitive development, results from the present study are the first to our knowledge to test and find support for a significant association between mindful parenting and child language development. In prior literature, mothers’ use of joint attention and sensitive responsiveness have been linked to children’s subsequent expressive and receptive language skills from 18 – 36 months (Barnett et al., 2012; Leigh et al., 2011). Joint attention requires that the mother *notices* what her child is attending to and responds accordingly. The mindful parenting measure in the present study included skills such as “pay[ing] close attention to your child when you are spending time together” and “really listening to your child.” This kind of attunement allows for greater joint attention between mother and child, as well as richer verbal exchanges that follow the child’s lead and support language development.

### Maternal mindful parenting: links to observed maternal responsivity and lack of punishment

Higher mindful parenting at 18 months was associated with higher observed 18-month maternal responsivity, controlling for symptoms of maternal depression. The maternal responsivity subscale measured mothers’ engagement with the child (e.g., “parent responds verbally to child’s vocalizations”) and affection toward the child (e.g., “parent caresses or kisses child at least once”). As both active attention toward the child and affection for the child are core components of mindful parenting, these findings provide support that mothers’ self-reports of mindful parenting are linked to their observed behaviors with their child.

Contrary to expectations, mindful parenting was not significantly associated with observed maternal lack of punishment toward the child. It may be that parents and children were on their “best behavior” in the presence of the experimenters, with children being less likely to test parental limits and parents less likely to engage in the punishing behaviors captured by the scale (e.g., shouting at child, spanking child, scolding child, expressing annoyance). Exploratory post hoc analyses adding the two observed parenting measures as predictors of child outcomes (along with mindful parenting and maternal depressive symptoms) indicated that maternal responsivity was associated with lower child internalizing symptoms and higher social competence, while maternal lack of punishment was associated with lower child externalizing symptoms. Thus, among the primarily Black and Latina mothers in this sample, the quick and cost-effective self-reported mindful parenting measure emerged as a more consistent predictor of child outcomes than self-reported depression and the two observed measures of maternal parenting.

### Mindful parenting and child outcomes: moderation by child negative emotionality

Consistent with our hypothesis, child NE significantly moderated the association between mindful parenting and both child externalizing symptoms and social competence. However, the pattern of moderation differed from our expectations. Consistent with the tenets of differential susceptibility (Belsky & Pluess, 2009), we hypothesized that maternal mindful parenting would have a particularly strong impact on child outcomes for children who were high on NE, based on these children being at higher risk for emotional and behavioral problems, and thus having more to gain from having a highly mindful parent. Instead, mindful parenting was most strongly associated with child externalizing symptoms and social competence for children a standard deviation below the mean on NE, and most weakly associated with these outcomes for children a standard deviation above the mean on NE. Possible scores on the NE measure used in the present study range from 0 – 26, with a mean of 7.95 (*SD* = 4.88) in the current sample. Thus, as reported by mothers, children in this sample tended to have NE in the mild to moderate range.

Overall, it seems mindful parenting did not have a strong enough effect to have a positive impact on child outcomes in the context of moderately high child NE. Prior research among low-income, urban families has found a similar pattern of results in terms of protective family factors and youth antisocial behaviors. Specifically, higher levels of family cohesion and healthy family structure have been associated with lower levels of antisocial adolescent behavior, but this protective effect was only evident for families living in neighborhoods characterized by low to moderate risk. Among families in the highest risk-neighborhoods, this association was not statistically reliable (Gorman-Smith et al., 2000; Shaw et al., 2004). It is possible that mindful parenting functions similarly, acting as a protective factor but only up to a certain threshold of risk. Future research is necessary to better understand the relationship between mindful parenting and child characteristics, including temperament.

### Maternal mindful parenting and depressive symptoms

Maternal depressive symptoms during early childhood are a robust and well-established predictor of child social – emotional and behavioral problems (Goodman et al., 2011; Trussell et al., 2018; Wall-Wieler et al., 2020). Thus, it is notable that mindful parenting was a significant predictor of all four child outcomes controlling for symptoms of maternal depression, and in the case of social competence and productive language, was a significant predictor of these outcomes while maternal depression was not. It is possible that some mothers may feel more comfortable reporting honestly about mindful parenting compared to symptoms of depression, particularly in the current sample. Concerns about mental health stigma tend to be stronger among low-income and ethnic minority women (Freed et al., 2012; Olfson et al., 2003). In particular, Latina mothers may be more likely to believe that mental health problems should not be shared outside of the family unit (Schraufnagel et al., 2006), whereas perceived pressure among Black women to be “strong” may interfere with acknowledging mental health problems (Nicolaidis & Raymaker, 2015; Nicolaidis et al., 2010). Future research is necessary to further elucidate the relationship between maternal depression and mindful parenting and whether this relationship differs as a function of cultural and sociodemographic factors.

### Limitations and future directions

The present study is the first to explore longitudinal associations between mindful parenting and child outcomes in early childhood within a racially and ethnically diverse U.S.-based sample. Strengths include the use of both observational and parent-reported measures and consideration of maternal depressive symptoms. Limitations should also be acknowledged. First, we used two subscales (i.e., *listening with full attention; emotional awareness of self and child;*10 items) from the 31-item Interpersonal Mindfulness in Parenting Scale (IM-P). The decision to include the abbreviated version of the IM-P was made by the Smart Beginnings study team based on the aims of the larger study from which the data were obtained. The 10-item measure was quick to administer and significantly predicted child outcomes. However, it also raises questions about whether other components of mindfulness included in the IMP (e.g., compassion for self and child; self-regulation in the parenting relationship) would similarly relate to child outcomes. More research is necessary to clarify if particular aspects of mindful parenting may be more important for certain developmental outcomes compared to others.

Second, although our self-report measures of depressive symptoms (EPDS) and child outcomes (CBCL) are widely used and have demonstrated high reliability and validity, there are possible shared reporter biases resulting from mothers reporting on their mindful parenting, symptoms of depression, and children’s behavior. Future research examining associations between mindful parenting and child outcomes would benefit from utilizing objective, observational measures of children’s behavior, and/or utilizing multiple reporters (e.g., father, teacher).

Third, families in this sample were recruited as a part of a randomized controlled trial focusing on parenting and children’s school readiness outcomes. It is therefore reasonable to wonder whether the intervention may have influenced our variables of interest. However, we controlled for intervention status in all analyses, and post hoc analyses indicated that within this sample, intervention families did not significantly differ from control families on any variables included in our analyses (Supplemental Table 5). Thus, we are confident that the intervention did not significantly influence our results.

Fourth, due to difficulties with scheduling and concerns about neighborhood safety, a majority (57%) of the 18-month assessments in New York took – and a smaller percentage of visits in Pittsburgh (i.e., 14%) – took place in the lab rather than in the family’s home. Families who completed their 18-month assessment in the lab were found to have higher levels of observed lack of punishment (i.e., less punishing behavior) compared to families who had their assessment in their home. Internal reliability was similar and adequate for both observed parenting measures across lab and home visits; families did not differ on any other predictor or demographic variables as a function of their visit location. As none of our significant findings involved the observed lack of punishment measure, we do not believe the visit location is a threat to the validity of our results. Still, additional research is needed to determine whether observed parenting practices as measured by the HOME may differ in a lab- versus home-based environment.

Fifth, more research is needed to examine the *mechanisms* by which maternal mindful parenting impacts early child outcomes. Based on prior research linking higher levels of mindful parenting to more positive observed parenting practices (e.g., higher levels of warmth, sensitivity (Duncan et al., 2015; Wang et al., 2018; Zeegers et al., 2019) and our finding an association between mindful parenting and higher maternal responsivity, parent – child interactions are a promising potential mediator of associations between mindful parenting and child outcomes. As mindful parenting was measured concurrently with observed responsivity and lack of punishment in this sample, our data were not appropriate for testing a mediation model. However, testing mediational processes would be a worthy goal of future research using longitudinal measurement. Finally, there are many parental characteristics besides depressive symptoms that could be theorized to associate with mindful parenting, including parental sense of efficacy, enjoyment in parenting, and symptoms of anxiety and other types of psychopathology. Future studies would benefit from exploring how other maternal *and paternal* factors may relate to mindful parenting.

Overall, results of the present study extend prior findings linking mindful parenting and child outcomes beyond the low-risk, non-Hispanic White, and primarily adolescent samples used in past research to a low-income sample of Black and Latina mothers and their young children. Although future studies both replicating and extending our findings are necessary, results provide strong preliminary evidence that maternal mindful parenting makes a contribution to child outcomes that is distinct from maternal depression. Our findings suggest that mindful parenting is important to consider as a protective factor in studies assessing early childhood risk for social – emotional and behavioral problems, and may be a worthwhile target for parent-focused interventions among Black and Latina mothers during early childhood.

## Supporting information

10.1017/S0954579425101004.sm001 Taraban et al. supplementary material Taraban et al. supplementary material

## Data Availability

Data are available upon reasonable request to the first author.

## Supplementary material

The supplementary material for this article can be found at https://doi.org/10.1017/S0954579425101004.

## Preregistration statement

This manuscript was not preregistered, due to it being secondary analysis outside the scope of the original aims of the associated grant.
