# Supplementary material for: Mindful mamas: Black and Latina mothers’ mindful parenting predicts toddlers’ later social–emotional and cognitive functioning
Source: Dev Psychopathol. 2025 Dec 17:1–15. Online ahead of print. doi: 10.1017/S0954579425101004 (PMC12825944; doi:10.1017/S0954579425101004)
Supplement: Taraban et al. supplementary material [file S0954579425101004sup001.docx]

**Supplemental Table 1**

*Aim 1 path analysis relations with and between demographic covariates.*

| **Path** | **β (SE)** | ***p*** |
| --- | --- | --- |
| Child Latinx 🡪 mindful parenting | .05 (.16) | .76 |
| Child Black 🡪 mindful parenting | .30 (.16) | .05**^+^** |
| Child other race 🡪 mindful parenting | .03 (.07) | .65 |
| **Child girl 🡪 mindful parenting** | **.12 (.05)** | **.03*** |
| Parental education 🡪 mindful parenting | -.04 (.06) | .56 |
| Child Latinx 🡪 maternal depressive sx. | -.33 (.18) | .07**^+^** |
| Child Black 🡪 maternal depressive sx. | -.17 (.18) | .34 |
| **Child other race 🡪 maternal depressive sx.** | **-.20 (.08)** | **.01*** |
| Child girl 🡪 maternal depressive sx. | .05 (.06) | .41 |
| Parental education 🡪 maternal depressive sx. | .05 (.06) | .37 |
| Child Latinx 🡪 maternal responsivity | .02 (.10) | .81 |
| **Child Black 🡪 maternal responsivity** | **-.21 (.10)** | **.03*** |
| Child other race 🡪 maternal responsivity | -.04 (.07) | .60 |
| Child girl 🡪 maternal responsivity | .00 (.06) | .94 |
| **Parental education 🡪 maternal responsivity** | **.22 (.05)** | **<.001***** |
| **Child Latinx 🡪 maternal lack of punishment** | **.45 (.11)** | **<.001***** |
| Child Black 🡪 maternal lack of punishment | -.12 (.11) | .27 |
| Child other race 🡪 maternal lack of punishment | .13 (.08) | .10 |
| Child girl 🡪 maternal lack of punishment | .04 (.05) | .37 |
| **Parental education 🡪 maternal lack of punishment** | **.11 (.05)** | **.04*** |
| Child Latinx 🡪 child internalizing | .17 (.20) | .39 |
| Child Black 🡪 child internalizing | -.08 (.20) | .69 |
| Child other race 🡪 child internalizing | -.02 (.09) | .87 |
| Child girl 🡪 child internalizing | -.04 (.05) | .39 |
| **Parental education 🡪 child internalizing** | **-.21 (.05)** | **<.001***** |
| Child Latinx 🡪 child externalizing | -.21 (.27) | .44 |
| Child Black 🡪 child externalizing | -.16 (.27) | .56 |
| Child other race 🡪 child externalizing | -.10 (.12) | .43 |
| **Child girl 🡪 child externalizing** | **-.10 (.05)** | **.04*** |
| Parental education 🡪 child externalizing | -.06 (.05) | .23 |
| Child Latinx 🡪 child social competence | .17 (.25) | .50 |
| Child Black 🡪 child social competence | .07 (.25) | .77 |
| Child other race 🡪 child social competence | .10 (.11) | .33 |
| **Child girl 🡪 child social competence** | **.12 (.06)** | **.03*** |
| **Parental education 🡪 child social competence** | **.18 (.06)** | **.002**** |
| Child Latinx 🡪 child language | -.10 (.18) | .57 |
| Child Black 🡪 child language | -.06 (.18) | .73 |
| Child other race 🡪 child language | .07 (.1) | .45 |
| Child girl 🡪 child language | .09 (.06) | .11 |
| Parental education 🡪 child language | -.01 (.06) | .89 |
| **Child Latinx 🡨🡪 child Black** | **-.87 (.03)** | **<.001***** |
| **Child Latinx 🡨🡪 child other race** | **-.19 (.03)** | **<.001***** |
| **Child Latinx 🡨🡪 parental education** | **-.37 (.05)** | **<.001***** |
| **Child Black 🡨🡪 child other race** | **-.21 (.03)** | **<.001***** |
| **Child Black 🡨🡪 parental education** | **.26 (.05)** | **<.001***** |
| **Child other race 🡨🡪 parental education** | **.18 (.06)** | **.003**** |

*Note.* **^+^***p* < .10, **p* < .05, ***p* < .01; ****p* < .001.

**Supplemental Table 2**

*Aim 2 path analysis relations with and between demographic covariates.*

| **Path** | **β (SE)** | ***p*** |
| --- | --- | --- |
| Child Latinx 🡪 mindful parenting | .05 (.16) | .75 |
| **Child Black 🡪 mindful parenting** | **.31 (.16)** | **.048*** |
| Child other race 🡪 mindful parenting | .03 (.07) | .65 |
| **Child girl 🡪 mindful parenting** | **.12 (.05)** | **.03*** |
| Parental education 🡪 mindful parenting | -.04 (.06) | .57 |
| Child Latinx 🡪 child negative emotionality | .02 (.13) | .85 |
| Child Black 🡪 child negative emotionality | .07 (.12) | .55 |
| Child other race 🡪 child negative emotionality | -.06 (.06) | .29 |
| **Child girl 🡪 child negative emotionality** | **-.12 (.06)** | **.02*** |
| Parental education 🡪 child negative emotionality | -.10 (.06) | .099**^+^** |
| Child Latinx 🡪 maternal depressive sx. | -.33 (.18) | .07**^+^** |
| Child Black 🡪 maternal depressive sx. | -.17 (.18) | .34 |
| **Child other race 🡪 maternal depressive sx.** | **-.20 (.08)** | **.01*** |
| Child girl 🡪 maternal depressive sx. | .05 (.06) | .42 |
| Parental education 🡪 maternal depressive sx. | .05 (.06) | .39 |
| Child Latinx 🡪 child internalizing | .17 (.16) | .28 |
| Child Black 🡪 child internalizing | -.11 (.16) | .51 |
| Child other race 🡪 child internalizing | .00 (.07) | .99 |
| Child girl 🡪 child internalizing | -.01 (.05) | .77 |
| **Parental education 🡪 child internalizing** | **-.16 (.05)** | **.001**** |
| Child Latinx 🡪 child externalizing | -.2 (.22) | .36 |
| Child Black 🡪 child externalizing | -.16 (.22) | .46 |
| Child other race 🡪 child externalizing | -.08 (.1) | .44 |
| Child girl 🡪 child externalizing | -.05 (.05) | .24 |
| Parental education 🡪 child externalizing | .00 (.04) | .95 |
| Child Latinx 🡪 child social competence | .15 (.23) | .52 |
| Child Black 🡪 child social competence | .05 (.23) | .83 |
| Child other race 🡪 child social competence | .09 (.1) | .35 |
| Child girl 🡪 child social competence | .10 (.06) | .08**^+^** |
| **Parental education 🡪 child social competence** | **.15 (.05)** | **.005**** |
| Child Latinx 🡪 child language | -.12 (.17) | .47 |
| Child Black 🡪 child language | -.08 (.17) | .62 |
| Child other race 🡪 child language | .07 (.09) | .48 |
| Child girl 🡪 child language | .07 (.06) | .19 |
| Parental education 🡪 child language | -.03 (.06) | .65 |
| **Child Latinx 🡨🡪 child Black** | **-.87 (.03)** | **<.001***** |
| **Child Latinx 🡨🡪 child other race** | **-.19 (.03)** | **<.001***** |
| **Child Latinx 🡨🡪 parental education** | **-.38 (.05)** | **<.001***** |
| **Child Black 🡨🡪 child other race** | **-.21 (.03)** | **<.001***** |
| **Child Black 🡨🡪 parental education** | **.27 (.05)** | **<.001***** |
| **Child other race 🡨🡪 parental education** | **.18 (.06)** | **.003**** |

*Note.* **^+^***p* < .10, **p* < .05, ***p* < .01; ****p* < .001.

**Supplemental Table 3**

*Post-hoc exploratory analysis, testing observed maternal responsivity and lack of punishment as predictors of child outcomes.*

| **Path** | **β (SE)** | ***p*** |
| --- | --- | --- |
| **Mindful parenting 🡪 child internalizing** | **-.13 (.06)** | **.04*** |
| **Mindful parenting 🡪 child externalizing** | **-.17 (.06)** | **<.01**** |
| **Mindful parenting 🡪 child social competence** | **.16 (.06)** | **<.01**** |
| **Mindful parenting 🡪 child language** | **.16 (.06)** | **.01*** |
| **Maternal responsivity 🡪 child internalizing** | **-.14 (.07)** | **.04*** |
| Maternal responsivity 🡪 child externalizing | -.07 (.06) | .25 |
| **Maternal responsivity 🡪 child social competence** | **.18 (.06)** | **<.001***** |
| **Mindful responsivity 🡪 child language** | **.12 (.05)** | **.03** |
| Maternal lack of punishment 🡪 child internalizing | -.02 (.06) | .79 |
| **Maternal lack of punishment 🡪 child externalizing** | **-.15 (.07)** | **.03*** |
| Maternal lack of punishment 🡪 child social competence | .02 (.07) | .79 |
| Mindful lack of punishment 🡪 child language | .06 (.07) | .43 |
| **Maternal depressive sx. 🡪 child internalizing** | **.29 (.07)** | **<.001***** |
| **Maternal depressive sx. 🡪 child externalizing** | **.34 (.06)** | **<.001***** |
| Maternal depressive sx. 🡪 child social competence | -.05 (.06) | .41 |
| Maternal depressive sx. 🡪 child language | .07 (.06) | .25 |
| **Mindful parenting 🡨🡪 maternal responsivity** | **.18 (.05)** | **<.001**** |
| Mindful parenting 🡨🡪 maternal lack of punishment | .06 (.06) | .31 |
| **Mindful parenting 🡨🡪 maternal depressive sx.** | **-.39 (.05)** | **<.001**** |
| Maternal responsivity 🡨🡪 maternal lack of punishment | .07 (.06) | .28 |
| Maternal responsivity 🡨🡪 maternal depressive sx. | .01 (.06) | .84 |
| Maternal lack of punishment 🡨🡪 maternal depressive sx. | -.12 (.06) | .06**^+^** |
| **Child internalizing 🡨🡪 child externalizing** | **.52 (.04)** | **<.001***** |
| Child internalizing 🡨🡪 child social competence | -.11 (.06) | .06**^+^** |
| **Child internalizing 🡨🡪 child language** | **-.14 (.05)** | **<.01**** |
| **Child externalizing 🡨🡪 child social competence** | **-.25 (.06)** | **<.001**** |
| **Child externalizing 🡨🡪 child language** | **-.13 (.06)** | **.02*** |
| **Child social competence 🡨🡪 child language** | **.36 (.05)** | **<.001***** |
| Child Latinx 🡪 mindful parenting | .05 (.16) | .76 |
| Child Black 🡪 mindful parenting | .30 (.15) | .05**^+^** |
| Child other race 🡪 mindful parenting | .03 (.07) | .65 |
| **Child girl 🡪 mindful parenting** | **.12 (.05)** | **.03*** |
| Parental education 🡪 mindful parenting | -.04 (.06) | .56 |
| Child Latinx 🡪 maternal depressive sx. | -.33 (.18) | .07**^+^** |
| Child Black 🡪 maternal depressive sx. | -.17 (.18) | .34 |
| **Child other race 🡪 maternal depressive sx.** | **-.20 (.08)** | **.01** |
| Child girl 🡪 maternal depressive sx. | .05 (.06) | .41 |
| Parental education 🡪 maternal depressive sx. | .05 (.06) | .37 |
| Child Latinx 🡪 maternal responsivity | .00 (.11) | .98 |
| **Child Black 🡪 maternal responsivity** | -.16 (.11) | .15 |
| Child other race 🡪 maternal responsivity | -.05 (.07) | .49 |
| Child girl 🡪 maternal responsivity | .03 (.06) | .65 |
| **Parental education 🡪 maternal responsivity** | **.21 (.05)** | **<.001***** |
| **Child Latinx 🡪 maternal lack of punishment** | **.48 (.11)** | **<.001***** |
| Child Black 🡪 maternal lack of punishment | -.10 (.11) | .36 |
| Child other race 🡪 maternal lack of punishment | .14 (.08) | .06**^+^** |
| Child girl 🡪 maternal lack of punishment | .04 (.05) | .39 |
| **Parental education 🡪 maternal lack of punishment** | **.10 (.05)** | **<.05*** |
| Child Latinx 🡪 child internalizing | .18 (.2) | .35 |
| Child Black 🡪 child internalizing | -.11 (.19) | .56 |
| Child other race 🡪 child internalizing | -.02 (.09) | .84 |
| Child girl 🡪 child internalizing | -.04 (.05) | .39 |
| **Parental education 🡪 child internalizing** | **-.18 (.06)** | **< .001***** |
| Child Latinx 🡪 child externalizing | -.14 (.26) | .59 |
| Child Black 🡪 child externalizing | -.20 (.27) | .46 |
| Child other race 🡪 child externalizing | -.08 (.12) | .51 |
| Child girl 🡪 child externalizing | -.09 (.05) | .06**^+^** |
| Parental education 🡪 child externalizing | -.03 (.05) | .60 |
| Child Latinx 🡪 child social competence | .16 (.24) | .51 |
| Child Black 🡪 child social competence | .11 (.24) | .64 |
| Child other race 🡪 child social competence | .11 (.10) | .29 |
| **Child girl 🡪 child social competence** | **.12 (.05)** | **.03*** |
| **Parental education 🡪 child social competence** | **.14 (.06)** | **.02*** |
| Child Latinx 🡪 child language | -.13 (.18) | .46 |
| Child Black 🡪 child language | -.03 (.17) | .86 |
| Child other race 🡪 child language | .07 (.10) | .46 |
| Child girl 🡪 child language | .09 (.06) | .12 |
| Parental education 🡪 child language | -.04 (.06) | .54 |
| **Child Latinx 🡨🡪 child Black** | **-.87 (.03)** | **< .001***** |
| **Child Latinx 🡨🡪 child other race** | **-.19 (.03)** | **< .001***** |
| **Child Latinx 🡨🡪 parental education** | -.37 (.05) | **< .001***** |
| **Child Black 🡨🡪 child other race** | -.21 (.03) | **< .001***** |
| **Child Black 🡨🡪 parental education** | .26 (.05) | **< .001***** |
| **Child other race 🡨🡪 parental education** | .18 (.06) | **< .001***** |

*Note.* Model fit: χ^2^ (9) = 9.79, *p* = .37; RMSEA = .02, 90% CI [.00, .07]; CFI = 1.00. **^+^***p* < .10, **p* < .05, ***p* < .01; ****p* < .001.

**Supplemental Table 4**

*Post-hoc exploratory analysis testing maternal depressive symptoms as a moderator of associations between mindful parenting and child outcomes.*

| **Path** | **β (SE)** | ***p*** |
| --- | --- | --- |
| Mindful parenting 🡪 child internalizing | -.05 (.06) | .44 |
| Mindful parenting 🡪 child externalizing | -.07 (.07) | .29 |
| **Mindful parenting 🡪 child social competence** | **.16 (.06)** | **.01*** |
| **Mindful parenting 🡪 child language** | **.15 (.07)** | **.02*** |
| **Child negative emotionality 🡪 child internalizing** | **.34 (.06)** | **<.001***** |
| **Child negative emotionality 🡪 child externalizing** | **.37 (.05)** | **<.001***** |
| **Child negative emotionality 🡪 child social competence** | **-.15 (.07)** | **.02** |
| Child negative emotionality 🡪 child language | -.11 (.06) | .07**^+^** |
| Mindful parenting x Maternal depressive sx. 🡪 child internalizing | .02 (.07) | .74 |
| Mindful parenting x Maternal depressive sx.🡪 child externalizing | .02 (.04) | .63 |
| Mindful parenting x Maternal depressive sx.🡪 child social competence | .05 (.06) | .41 |
| Mindful parenting x Maternal depressive sx.🡪 child language | -.03 (.06) | .59 |
| **Maternal depressive sx. 🡪 child internalizing** | **.21 (.06)** | **<.001***** |
| **Maternal depressive sx. 🡪 child externalizing** | **.28 (.06)** | **<.001***** |
| Maternal depressive sx. 🡪 child social competence | -.02 (.06) | .73 |
| Maternal depressive sx. 🡪 child language | .11 (.07) | .13 |
| **Mindful parenting 🡨🡪 child negative emotionality** | **-.36 (.06)** | **<.001***** |
| **Mindful parenting 🡨🡪 maternal depressive sx.** | **-.39 (.05)** | **<.001***** |
| **Child negative emotionality 🡨🡪 maternal depressive sx.** | **.29 (.06)** | **<.001***** |
| **Child internalizing 🡨🡪 child externalizing** | **.45 (.04)** | **<.001***** |
| Child internalizing 🡨🡪 child social competence | -.10 (.05) | .07**^+^** |
| **Child internalizing 🡨🡪 child language** | **-.13 (.05)** | **.01*** |
| **Child externalizing 🡨🡪 child social competence** | **-.24 (.06)** | **<.001***** |
| **Child externalizing 🡨🡪 child language** | **-.11 (.06)** | **<.05*** |
| **Child social competence 🡨🡪 child language** | .36 (.05) | **<.001***** |
| Child Latinx 🡪 mindful parenting | .04 (.16) | .79 |
| **Child Black 🡪 mindful parenting** | **.31 (.16)** | **<.04** |
| Child other race 🡪 mindful parenting | .03 (.07) | .65 |
| **Child girl 🡪 mindful parenting** | **.11 (.05)** | **<.04** |
| Parental education 🡪 mindful parenting | -.03 (.06) | .61 |
| Child Latinx 🡪 child negative emotionality | .03 (.13) | .84 |
| Child Black 🡪 child negative emotionality | .07 (.12) | .59 |
| Child other race 🡪 child negative emotionality | -.06 (.06) | .28 |
| **Child girl 🡪 child negative emotionality** | **-.12 (.06)** | **.03*** |
| Parental education 🡪 child negative emotionality | -.11 (.06) | .08**^+^** |
| Child Latinx 🡪 maternal depressive sx. | -.32 (.18) | .07**^+^** |
| Child Black 🡪 maternal depressive sx. | -.17 (.19) | .33 |
| **Child other race 🡪 maternal depressive sx.** | **-.20 (.08)** | **.01** |
| Child girl 🡪 maternal depressive sx. | .05 (.06) | .40 |
| Parental education 🡪 maternal depressive sx. | .05 (.06) | .42 |
| Child Latinx 🡪 child internalizing | .15 (.16) | .34 |
| Child Black 🡪 child internalizing | -.14 (.16) | .38 |
| Child other race 🡪 child internalizing | -.01 (.07) | .95 |
| Child girl 🡪 child internalizing | -.02 (.05) | .68 |
| **Parental education 🡪 child internalizing** | **-.17 (.05)** | **<.001***** |
| Child Latinx 🡪 child externalizing | -.25 (.23) | .29 |
| Child Black 🡪 child externalizing | -.23 (.23) | .34 |
| Child other race 🡪 child externalizing | -.09 (.11) | .41 |
| Child girl 🡪 child externalizing | -.07 (.05) | .13 |
| Parental education 🡪 child externalizing | -.01 (.05) | .82 |
| Child Latinx 🡪 child social competence | .17 (.24) | .49 |
| Child Black 🡪 child social competence | .08 (.24) | .73 |
| Child other race 🡪 child social competence | .10 (.10) | .36 |
| Child girl 🡪 child social competence | .10 (.06) | .06**^+^** |
| **Parental education 🡪 child social competence** | **.14 (.06)** | **.01*** |
| Child Latinx 🡪 child language | -.09 (.17) | .60 |
| Child Black 🡪 child language | -.04 (.17) | .82 |
| Child other race 🡪 child language | .07 (.09) | .43 |
| Child girl 🡪 child language | .08 (.06) | .17 |
| Parental education 🡪 child language | -.03 (.06) | .65 |
| **Child Latinx 🡨🡪 child Black** | **-.87 (.03)** | **<.001***** |
| **Child Latinx 🡨🡪 child other race** | **-.19 (.03)** | **<.001***** |
| **Child Latinx 🡨🡪 parental education** | **-.38 (.05)** | **<.001***** |
| **Child Black 🡨🡪 child other race** | **-.22 (.03)** | **<.001***** |
| **Child Black 🡨🡪 parental education** | **.27 (.05)** | **<.001***** |
| **Child other race 🡨🡪 parental education** | **.18 (.06)** | **<.001***** |

*Note.* Model fit: χ^2^ (18) = 38.50, *p* < .01; RMSEA = .06, 90% CI [.03, .09]; CFI = .96. **^+^***p* < .10, **p* < .05, ***p* < .01; ****p* < .001.

**Supplemental Table 5.**

*Comparisons on demographic and primary variables between families randomly assigned versus not assigned to the intervention condition.*

| **Measure** | **Child Age** | **Mean(SD) or *%*** | | **Comparison** |
| --- | --- | --- | --- | --- |
|  |  | **Control (*n* = 162)** | **Treatment (*n* = 149)** |  |
| Child Latinx | 6 mo. | 45% | 42% | χ^2^ (1) = 0.24, *p* = .62 |
| Child Black | 6 mo. | 48% | 50% | χ^2^ (1) = 0.15, *p* = .70 |
| Child White | 6 mo. | 2% | 3% | χ^2^ (1) = 0.70, *p* = .40 |
| Child Other Race | 6 mo. | 5% | 4% | χ^2^ (1) = 0.15, *p* = .70 |
| Child Female | 6 mo. | 50% | 41% | χ^2^ (1) = 2.31, *p* = .13 |
| Maternal Education | 6 mo. | 7.80 (2.53) | 8.34 (2.57) | *t*(310) = -1.88, *p* = .06 |
| Maternal Mindful Parenting | 18 mo. | 3.26 (0.55) | 3.29 (0.47) | *t*(314) = -0.48, *p* = .63 |
| Maternal Depressive Sx | 18 mo. | 4.15 (4.21) | 3.68 (3.99) | *t*(308) = 1.01, *p* = .31 |
| Maternal Responsivity | 18 mo. | 9.75 (1.96) | 9.86 (1.61) | *t*(305) = -0.54, *p* = .59 |
| Maternal Lack of punishment | 18 mo. | 5.00 (1.69) | 4.84 (1.74) | *t*(296) = 0.82, *p* = .41 |
| Child Negative Emotionality | 18 mo. | 7.96 (4.88) | 7.93 (4.90) | *t*(313) = 0.48, *p* = .94 |
| Child Internalizing Symptoms | 24 mo. | 9.51 (7.05) | 8.72 (6.30) | *t*(285) 0.99, *p* = .32 |
| Child Externalizing Symptoms | 24 mo. | 12.03 (8.34) | 11.88 (7.36) | *t*(286) = -0.16, *p* = .88 |
| Child Social Competence | 24 mo. | 44.12 (9.20) | 45.63 (8.30) | *t*(286) = -1.46, *p* = .15 |
| Child Language | 24 mo. | 35.07 (24.82) | 35.19 (24.23) | *t*(284) = -0.04, *p* = .97 |
